# At what stage in the undergraduate curriculum is it best to train in family medicine? A study from two medical schools in Spain

**DOI:** 10.1080/13814788.2019.1580264

**Published:** 2019-04-02

**Authors:** Mónica López-García, María Candelaria Ayuso-Raya, Jesús López-Torres-Hidalgo, Julio Montoya-Fernández, Francisco Campa-Valera, Francisco Escobar-Rabadán

**Affiliations:** a Healthcare Centre, Zone IV, Healthcare Service of Castilla-La Mancha (SESCAM), Albacete, Spain;; b Emergency Service of the Albacete General Hospital, SESCAM, Albacete, Spain;; c Healthcare Centre, Zone VIII, Albacete (SESCAM), Albacete, Spain;; d Albacete Medical School, Albacete, Spain;; e Primary Health Care in Albacete, SESCAM, Albacete, Spain;; f Don Paulino García Donas Healthcare Centre, Healthcare Service of Andalucía (SAS), Sevilla, Spain;; g Sevilla Medical School, Sevilla, Spain;; h Healthcare Centre, Zone IV, SESCAM, Albacete, Spain

**Keywords:** Family practice, internship and residency, medical education, medical schools, medical undergraduate, students

## Abstract

**Background:** A course in family medicine (FM) could dispel the possibility of negative stereotyping about this speciality, and instil in students a greater interest. However, when is it preferable: at the beginning or at the end of undergraduate training?

**Objectives:** To determine changes in knowledge and attitudes towards FM by medical students completing a course in primary care at the beginning or the end of the undergraduate training and whether those changes anticipate the choice of speciality.

**Methods:** Students from Albacete and Seville medical schools (primary care course in second and sixth years, respectively) were asked to respond to the ‘valuation of attitudes towards and knowledge of family medicine questionnaire’ (CAMF). Students from Albacete answered before and after the course, and in Seville second-year students answered at the end of the first trimester. All students were invited to respond again at the end of their undergraduate training. Afterwards, we investigated the score on the speciality exam (order for the election from highest to lowest score) and their choice of speciality. The outcome measures were the MIR exam score, the number in the ranking, the chosen speciality and the result of the CAMF.

**Results:** In Albacete 88 and 64 and in Seville 50 and 98 students responded in their second and sixth years, respectively. In Albacete, mean CAMF scores were 15.4, 22.7 before and after the course, and 21.8 at the end while in Seville, 13.9 in the second year, and 23.5 in the sixth year. Logistic regression analysis showed an association of the choice of FM only with the score on the speciality exam (OR: 0.667; 95%CI: 0.553–0.806).

**Conclusion:** There were no significant differences between CAMF scores at the end of undergraduate training. Only the score on the speciality exam predicts FM choice: the higher the score, the lower the probability of choosing FM.

KEY MESSAGES

- Spanish students show great interest in family medicine after a primary care course in their second year.
- Ending the undergraduate training, there are not significant differences with students who take the course in the sixth year.
- No differences have been found related to the choice of speciality between both groups.

## Introduction

The widely known cold climate towards primary care in medical academia constitutes a barrier to choosing this discipline as a career option [1]. In a previous study [2], we determined that students at the Albacete Medical School showed a remarkable initial lack of knowledge and poor opinion of family medicine (FM) and primary care, especially when compared with those of other countries [3]. We also demonstrated an improvement in the knowledge of and attitudes towards FM after completing a course in primary care. As it is well known, the experience of students during their clinical clerkship has a significant impact on their attitudes towards the speciality [4]. Within an international context of the growing demand for primary care physicians, this topic becomes of special interest [5]. As Schneider et al., pointed out [6], rigorous research is needed to assess how best to utilize limited educational resources to ensure that all students graduate with a core FM competence and increase matriculation into FM residency.

This way, a course in FM contributes to dispel possible negative stereotyping about the speciality and instils in students a higher interest. However, when is it preferable: at the beginning or the end of the undergraduate training? This question seems especially relevant in the Spanish context, where there has not been a tradition of undergraduate training in FM, and its introduction in medical schools is relatively recent (from the 1990s).

Primary care training in pre-clinical stages contributes to a better clinical performance by the students because it can help medical students to consolidate and integrate both the fundamental cognitive and clinical skills they will apply during the clinical years of medical training [7]. Dornan et al., pointed out that early experience motivates and satisfies students, helps them acclimatize to clinical environments and develop professionally [8]. However, for other authors, the attitude towards FM is even more favourable when medical students are ending their undergraduate years. This may be partially explained by the greater contact with family doctors [9].

The objectives of this study were: (1) to determine changes in the knowledge of and attitudes towards FM following a primary care course; (2) to know possible differences in the changes when the course is taken at the beginning or at the end of the undergraduate training; and (3) to examine if these changes predict the choice of FM as a speciality.

## Methods

This is a cohort study. In 2009–2010 academic year we selected students enrolled in their second year from two medical schools, both with a six-year curriculum. At the Albacete Medical School, students taking a primary care course were requested to respond to a self-administered, anonymous questionnaire before starting it, and the day of the final exam for that course. At Seville Medical School, students from the Valme Campus were requested to respond at the end of the first trimester. In their curriculum, the primary care course was in the sixth year. At both sites, the students were invited to answer the same questionnaire again at the end of the undergraduate training; that is, students enrolled in their sixth year in 2013–2014 academic year (in Seville not only Valme but also all of its campuses, because at that time we had the possibility also to contact the students of the other campuses). Students from Albacete Medical School responded to a ‘pencil and paper’ format in classrooms and the students from Seville Medical School responded in some cases with ‘pencil and paper’ and in other cases online. We registered the exam scores, the number in the ranking (number one is who gets the best score, number two for the second best, and so on) and the specialities chosen by the students after the exam to access the specialist medical training (MIR is the Spanish acronym) in 2015 and 2016, based on the information provided by the Ministry of Health on its website.

### Ethics

Ethics approval for this study was granted by the Investigation and Clinical Ethics Committee of the Albacete Area. Student participation in the study was voluntary. To compare related samples when it is appropriate, we asked the students for a reference number, proposing the four last digits of their National Identity Document, as this would be easy to remember. In any event, we guaranteed the anonymity of their responses.

### Study variables

The outcome measures were the MIR exam score, the number in the ranking, the chosen speciality and the result of the abbreviated version of the ‘valuation of attitudes towards and knowledge of family medicine questionnaire’ (CAMF is the Spanish acronym), a tool developed and validated by our group [10]. It consists of 21 closed response items, with five response options on a Likert scale ranging from ‘completely disagree’ to ‘completely agree’. The questionnaire also contained items on the socio-demographic and academic characteristics of the students: age, sex, population density of their town, social class estimated according to the Domingo and Marcos classification (based on the occupation of the parents) [11], number of subjects still pending (that is, the subjects that the student has not yet approved) and grade on entry to medical school (it depends on the university access test and the average high school grade point). The collected data was coded and entered into a computerized database using the SPSS 19.0 statistical programme.

### Statistical analysis

We analysed the responses to the items and calculated the overall score of the questionnaire, giving the following values: ‘completely disagree’: −2; ‘disagree’: −1; ‘indifferent’: 0; ‘agree’: +1; and ‘completely agree’: +2. To make the ‘−2’ value always correspond to the most unfavourable option regarding FM and +2 to the most favourable, we recoded the responses to items 10 and 15 with inverted scales.

The statistical analysis included a description of the different variables and a comparison of the groups of students (Pearson chi-squared test, Student’s *t*-test/nonparametric tests). We evaluated the students’ level of knowledge and their attitudes at the second and sixth years. We used the Wilcoxon signed-rank sum test to evaluate the statistical significance of the possible changes in scores for the different items. In addition, we calculated the effect size for each item [12]. The paired analysis was not possible for Seville students because of the poor registration of the reference number, so we compared their responses by the Mann–Whitney test as independent samples. Likewise, the questionnaire responses of the Albacete students were compared with those of the Seville students using the Mann–Whitney test as well as the effect size for each item. The association of choosing FM with other conditioning factors was determined by logistic regression.

## Results

Before the primary care course classes started, 74 students from Albacete (84.1% of those enrolled) completed the questionnaire. After taking the final exam at the end of the year, 87 students answered (98.9%), 73 of whom had responded to both questionnaires. At the Seville Medical School, 50 students (76.9%) completed the questionnaire in their second year. At the end of the sixth year, 64 students from Albacete and 98 from Seville answered. Table 1 sets out the socio-demographic and academic characteristics of the participants in the study. Students from both medical schools were very similar in their socio-demographic characteristics in the second year. However, they differed in their academic characteristics, as students from Albacete had significantly higher grades on entry to the medical school and there were a higher proportion of those without subjects pending. In the sixth grade they became quite similar regarding subjects pending; however, there were some differences in the socio-demographic characteristics, as among students from Seville the proportion of women had increased, they were somewhat older, and more were of lower middle and working class demographic.

**Table 1.** Socio-demographic and academic characteristics of the students participating in the study (in brackets per cent with relation to total who responded).

	Second year			Sixth year		
	Albacete	Seville	*P*	Albacete	Seville	*P*
Age: median (IR)	19 (19–20)	19 (19–19)	NS	23 (23–24)	24 (23–25)	0.003
Gender						
Female	51 (57.9)	36 (72.0)	NS	34 (53.1)	72 (73.5)	0.008
Man	37 (42.1)	14 (28.0)		30 (46.9)	26 (26.5)	
Number of inhabitants in their town						
<10 000	16 (18.6)	9 (18.7)		11 (17.2)	20 (20.4)	
10 000–30 000	10 (11.6)	11 (22.9)	NS	9 (14.1)	23 (23.5)	NS
30 001–100 000	19 (22.1)	7 (14.6)		15 (23.4)	17 (17.3)	
>100 000	41 (47.7)	21 (43.8)		29 (45.3)	38 (38.8)	
Not given	2	2		0	0	
Social class based on occupation						
Upper and upper middle	49 (56.3)	23 (46.0)		34 (54.0)	56 (59.6)	
Middle	37 (42.5)	18 (36.0)	NS	29 (46.0)	23 (24.5)	<0.001
Lower middle and low	1 (1.1)	9 (18.0)		0 (0.0)	15 (15.9)	
Not given	1	0		1	4	
Subjects pending						
0	79 (89.8)	36 (72.0)		46 (74.2)	72 (74.2)	
1	3 (3.4)	5 (10.0)	0.007	4 (6.5)	9 (9.3)	NS
≥2	6 (6.8)	9 (18.0)		12 (19.3)	16 (16.5)	
Not given	0	0		2	1	
Median grade at entry to medical school; maximum: 10 (IR)	9.14 (8.99–9.32)	8.61 (8.40–8.89)	<0.001	9.14 (8.94–9.30)	8.56 (8.34–8.90)	<0.001

IR: interquartile range; NS: no statistically significant differences.

In Albacete, mean CAMF scores were 15.4 and 22.7, before starting and after finishing the primary care course, respectively, and 21.8 at the end of the degree. In Seville, mean CAMF scores were 13.9 in the second year and 23.5 in the sixth year. There was a high similarity in the answers for the CAMF items in the second year among students of Albacete before studying the primary care subject and the students from Seville, without statistically significant differences in their CAMF scores. After completing the primary care course, students from Albacete showed a significant (*p* <0.001) improvement in the CAMF mean score, with more favourable answers to FM in almost all the items.

In the sixth year, CAMF scores were significantly (*P* < 0.001) higher than in the second year, both for students from Seville and for students from Albacete before taking the primary care course. Students from Seville showed more favourable responses to FM for most of the items in the sixth year compared to the second. There were no statistically significant differences in the CAMF score between the students from Albacete and Seville in the sixth year.

Table 2 sets out the socio-demographic and academic characteristics of the postgraduates who chose FM or another speciality. Students from Seville had a significantly higher probability (*P* < 0.0001) to choose FM than those from Albacete. The average age of those who chose FM was significantly higher (*P* = 0.003) than those who chose another speciality. Their grade at the entry to medical school was significantly lower (*P* = 0.008) and they were more likely to have subjects pending in the sixth year (*P* = 0.007). Those who chose FM had a lower score on the MIR exam and a higher number in the ranking; both differences were statistically significant (*P* < 0.001).

**Table 2.** Socio-demographic and academic characteristics of the postgraduates who chose FM or another specialty.

	Family medicine	Other specialty	*P*
Age: median (IR)	24 (23–25.25)	23 (23–24)	0.003
Gender			
Female	23 (67.6)	57 (60.6)	NS
Man	11 (32.4)	37 (39.4)	
Number of inhabitants in their town			
<10 000	2 (28.6)	11 (18.6)	NS
10 000–30 000	1 (14.3)	8 (13.6)	
30 001–100 000	0 (0.0)	15 (25.4)	
>100 000	4 (57.1)	25 (42.4)	
Not given	27	35	
Social class based on occupation			
Upper and upper middle	2 (28.6)	32 (53.3)	
Middle	4 (57.1)	28 (46.7)	NS
Lower middle and low	1 (14.3)	0 (0.0)	
Not given	27	34	
Subjects pending (sixth year)			
0	24 (70.6)	75 (83.3)	
1	6 (17.6)	3 (3.3)	0.007
≥2	4 (11.8)	5 (13.4)	
Not given	0	4	
Median grade at entry to medical school; maximum: 10 (IR)	8.62 (8.40–9.10)	9.14 (8.98–9.37)	0.008
Medical school			
Albacete	4 (11.8)	58 (61.7)	<0.0001
Seville	30 (88.2)	36 (38,3)	
CAMF score at the second year: mean (SD)	10.8 (5.1)	15.9 (6.7)	NS
CAMF score at the sixth year: mean (SD)	23.4 (5.6)	22.5 (6.8)	NS
MIR exam score: mean (SD)	51.69 (8.16)	75.66 (8.85)	<0.001
MIR exam ranking: median (IR)	5989.5 (5227–7072.5)	1609 (646.5–3006.75)	<0.001

IR: interquartile range; CAMF: valuation of attitudes towards and knowledge of family medicine questionnaire; MIR: the exam to access specialist medical training; NS: no statistically significant differences.

**Figure 1.**
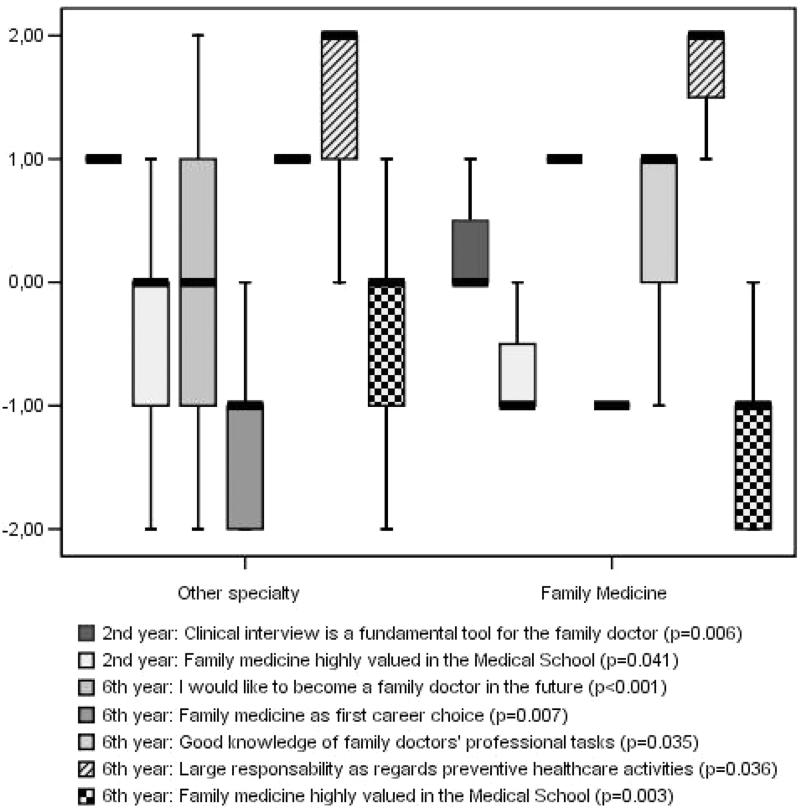
Median and interquartile range for score of the items with statistically significant differences when comparing postgraduates who chose family medicine or other specialty.

The answers to the different items at different times were compared for those who chose FM or another speciality. Figure 1 shows median and interquartile ranges for the score of the items that showed statistically significant differences.

The logistic regression analysis showed that only the order number in the MIR exam (OR: 1.002; 95%CI: 1.001–1.003) and the score in the exam (OR: 0.667; 95%CI: 0.553–0.806) were independent predictors for the choice of FM.

## Discussion

### Main findings

The interest in FM, which students from Albacete showed after completing a course in primary care at their second year, decreased at the end of the undergraduate training. At that moment, there were no significant differences between the CAMF scores for students enrolled in the primary care course at the second or sixth year. No differences were found in relation to the choice of speciality between both groups. In fact, only the score in the MIR exam predicts the choice of FM, so that the lower the score, the greater the probability of choosing it.

### Strengths and limitations

Results of this study seemed to be consistent with previous findings [13]. This work contributes to the many studies that have been published on the interest of medical students in FM with the strength of a prospective study, following-up from two cohorts of students in the second year and sixth years as well as after speciality election.

Our study, however, has some limitations. The fact that primary care teachers handed out the questionnaire to students might have biased the study through having a positive influence on the answers. Another possible limitation is that the questionnaires were applied immediately after their examination at Albacete Medical School, which may have led students to respond more positively than they would have done in other situations. We were aware of this potential limitation as well as the fact that students from Seville Medical School responded in some cases with ‘pencil and paper’ and in other instances online but we took this path because of feasibility issues.

### Training in pre-clinical stages of the curriculum

Many medical schools include primary care training in pre-clinical stages of the curriculum in the same way as the medical school of Albacete and it has been demonstrated that such training has contributed to a better clinical performance by the students. Along these lines, Musham and Chessman demonstrated in a study using a qualitative strategy that a strong negative image of FM existed amongst students and that a clerkship in FM dispelled this negative stereotyping and instilled in students greater respect for and interest in it [14]. Some comments indicated that students were favourably impressed by the philosophy of FM, emphasizing patient-focused care, prevention and the psychosocial emphasis.

### Training in clinical stages of the curriculum

However, many other medical schools include primary care training in the second half of the undergraduate training as the Seville Medical School. Dixon et al., conducted a qualitative study in the Hong Kong University medical school after the students had completed a part-time clerkship in FM for five weeks following completion of their academic studies [15]. The students’ impressions of FM before the clerkship were almost entirely negative with strong stereotypical views. Many of the students had not seriously considered FM as a career option. Clerkship in FM offered a contrast to their previous experience in hospital-based teaching. They found that FM was not as simple as they had thought, as it requires a specific skill set. These clerkships can have a long-term positive effect on attitudes towards FM as demonstrated by a study previously carried out by one of the authors of this group [16].

In Europe, most clinical general practice (GP)/FM rotations were placed in years four, five or six. Exceptionally, in some universities it involves the years one to six, which most likely has a positive influence on recruitment to GP/FM as students are exposed to positive role models throughout their entire education [17].

### Other factors affecting career choice: implications for future research

Many factors have been proposed in the international literature affecting career choice toward FM. Puertas et al., developed a comparison framework with common and specific factors that influence career choice in primary care among medical students from high-, middle- and low-income countries [18]. Several factors common to all countries were identified. Facilitators were exposure to rural location, role models, and working conditions. Barriers were low income, prestige, and medical school environment. Some factors specific to middle- and low-income countries were the understanding of rural needs and intellectual challenge. Other factors specific to high-income countries were the attitude towards social problems, voluntary work, influence of family, and length of residency. Scott et al., collected data from 1,542 students in Canadian medical schools at entry level and followed them prospectively to their residency choice [19]. The following entry variables predicted whether a student named FM as top residency choice: being older; being engaged or in a long-term relationship; not having parents with postgraduate university education nor having family or close friends practicing medicine; having undertaken voluntary work in a developing nation; not volunteering with elderly people; desire for varied scope of practice; a societal orientation; a lower interest in research; desire for short postgraduate training; and lower preference for medical versus social problems.

However, it is not only the students’ characteristics that influence this choice; in particular, we have to consider that the prevailing primary care culture at school also plays a role [20]. Trainees report a decreased number of primary care faculty role models who are satisfied with, and who find meaning in their work. Trainees see primary care physicians struggling with limited time to care for complex patients, overwhelming administrative or documentation burdens, and having to answer phone calls and secure e-mails on top of an already full workday [21].

As Naimer et al., pointed out [22], it is complicated to attract students to an unattractive speciality. Students who are not interested in FM more often perceive FM as being a boring speciality and as neither being prestigious or as an affordable academic opportunity.

All these variables need to be considered and researched further. 

## Conclusion

The interest of Spanish medical students in FM increased after completing a course in primary care but there are no significant differences at the end of the undergraduate training for students who were enrolled in the primary care course at the second or sixth year. No differences have been found related to the choice of speciality between either group.

## Supplementary Material

Appendix 1

Appendix 2
