# Supplementary material for: At what stage in the undergraduate curriculum is it best to train in family medicine? A study from two medical schools in Spain
Source: Eur J Gen Pract. 2019 Apr 2;25(2):91–7. doi: 10.1080/13814788.2019.1580264 (PMC6493302; doi:10.1080/13814788.2019.1580264)
Supplement: Appendix 1 [file IGEN_A_1580264_SM7378.doc]

Appendix 1.- Median (interquartile range) for each of the CAMF items. Statistical significance and the effect size for each item for different comparisons between groups is shown in Appendix 2.

| Item | Albacete | | | Sevilla | |
| --- | --- | --- | --- | --- | --- |
|  | A1 | A2 | A3 | S1 | S2 |
| 1. I would like to become a family doctor in the future | 0  (-1, 1) | 0  (-1, 1) | 0  (-1, 1) | -0.5  (-1, 0) | 1 (0, 1) |
| 2. Potential of family medicine to improve the health of the community | 1 (1, 2) | 1 (1, 2) | 2 (1, 2) | 1 (1, 2) | 2 (2, 2) |
| 3. Better healthcare compared to the previous ambulatory system | 1 (0, 1) | 2 (1, 2) | 1 (1, 1) | 0 (0, 1) | 1 (0, 1) |
| 4. Family medicine as first career choice | -1  (-2, -1) | -1  (-1, 0) | -1  (-2, -1) | -2  (-2, -1) | -1  (-2, -1) |
| 5. Responsibility of the family doctor for the health of the community | 1 (1, 2) | 1 (1, 2) | 1.5  (1, 2) | 1 (1, 2) | 2 (1, 2) |
| 6. Team work improves medical care | 2 (1, 2) | 2 (1, 2) | 2 (1, 2) | 1 (1, 2) | 2 (1, 2) |
| 7. Good knowledge of family doctors’ professional tasks | 0.5  (0, 1) | 1 (1, 2) | 1 (0, 1) | 0  (-0.25, 1) | 1 (1, 1) |
| 8. Primary care is the first medical contact with the healthcare system | 1 (1, 2) | 2 (2, 2) | 2 (1, 2) | 1  (0, 1.25) | 2 (1, 2) |
| 9. Clinical interview is a fundamental tool for the family doctor | 1 (1, 2) | 2 (1, 2) | 2 (2, 2) | 1  (1, 1.25) | 2 (2, 2) |
| 10. Family doctors manage health problems of little importance | 1 (0, 1) | 1 (0, 1) | 1  (1,1.75) | 1 (0, 1) | 2 (1, 2) |
| 11. Continual improving quality of care is a main objective | 1 (1, 1) | 1 (1, 2) | 1 (1, 2) | 1 (1, 2) | 2 (1, 2) |
| 12. Large responsibility as regards preventive healthcare activities | 1 (1, 2) | 2 (1, 2) | 2 (1, 2) | 1 (1, 2) | 2 (2, 2) |
| 13. Family doctors must have excellent communication skills | 1 (1, 2) | 2 (1, 2) | 1 (1, 2) | 1 (1, 2) | 2 (1, 2) |
| 14. Family medicine highly valued in the Faculty | 0  (-1, 0) | 0  (-1, 1) | 0  (-1, 0) | 0  (-1, 0) | -1  (-2, -1) |
| 15. Family medicine is not a very intellectually stimulating speciality | 0  (-1, 1) | 0 (0, 1) | 1 (0, 1) | 0  (-1, 1) | 1 (0, 1) |
| 16. Family doctors have a large work overload | 1  (0.75,1) | 1 (1, 2) | 1 (1, 2) | 1 (1, 2) | 2 (1, 2) |
| 17. A course in primary care in the medical school is appropriate | 1 (1, 2) | 1 (1, 2) | 1 (1, 2) | 1 (1, 2) | 2 (1, 2) |
| 18. Low efficiency of a health system directed exclusively to diagnosis and treatment | 0 (0, 1) | 1 (1, 2) | 1  (0.25,1) | 1 (0, 1) | 1 (0, 2) |
| 19. Family doctors should provide comprehensive and continuing healthcare | 1 (1, 2) | 2 (1, 2) | 2 (1, 2) | 2 (1, 2) | 2 (1, 2) |
| 20. The family doctor is clinically competent to provide most of the health care an individual may require | 1 (0, 1) | 1  (1, 1.5) | 1 (1, 2) | 1 (0, 1) | 1 (1, 2) |
| 21. Family doctors provide health care at their surgeries and at the patient’s home | 1 (0, 1) | 1 (1, 2) | 1 (1, 2) | 0 (0, 1) | 1 (1, 2) |

A1: Albacete, 2^nd^ at the beginning; A2: Albacete, 2^nd^ at the end; A3: Albacete, 6^th^;

S1: Sevilla, 2^nd^ ; S2: Sevilla, 6^th^
