# Supplementary material for: At what stage in the undergraduate curriculum is it best to train in family medicine? A study from two medical schools in Spain
Source: Eur J Gen Pract. 2019 Apr 2;25(2):91–7. doi: 10.1080/13814788.2019.1580264 (PMC6493302; doi:10.1080/13814788.2019.1580264)
Supplement: Appendix 2 [file IGEN_A_1580264_SM7367.doc]

Appendix 2.- Statistical significance and the effect size (ES) for each of the CAMF items, for different comparisons between groups scores shown in Appendix 1. Cells in grey correspond to values of p>0.05 and small or negligible practical importance effect size values.

| Item | A1 vs S1 | | A1 vs A2 | | A1 vs A3 | | A2 vs A3 | | S1 vs S2 | | A3 vs S2 | |
| --- | --- | --- | --- | --- | --- | --- | --- | --- | --- | --- | --- | --- |
|  | p  p  2 | ES | p  2 | ES | p | ES | p | ES | p | ES | p | ES |
| 1 | 0.044 | 0.38 | 0.028 | 0.23 | NS | 0.01 | NS | 0.22 | <0.001 | 0.88 | 0.009 | 0.45 |
| 2 | NS | 0.08 | <0.001 | 0.53 | <0.001 | 0.62 | NS | 0.10 | <0.001 | 0.86 | 0.001 | 0.40 |
| 3 | 0.009 | 0.44 | <0.001 | 1.00 | NS | 0.31 | <0.001 | 0.86 | 0.01 | 0.38 | 0.028 | 0.37 |
| 4 | NS | 0.32 | <0.001 | 0.58 | NS | 0.04 | 0.001 | 0.46 | 0.016 | 0.50 | NS | 0.17 |
| 5 | 0.024 | 0.44 | 0.013 | 0.25 | 0.012 | 0.43 | NS | 0.25 | NS | 0.26 | NS | 0.24 |
| 6 | NS | 0.30 | NS | 0.08 | NS | 0.16 | NS | 0.08 | 0.001 | 0.53 | NS | 0.14 |
| 7 | 0.044 | 0.37 | <0.001 | 0.90 | <0.001 | 0.71 | NS | 0.23 | <0.001 | 0.77 | NS | 0.26 |
| 8 | 0.009 | 0.46 | <0.001 | 0.65 | 0.002 | 0.47 | NS | 0.27 | <0.001 | 0.74 | NS | 0.10 |
| 9 | NS | 0.06 | <0.001 | 0.85 | <0.001 | 1.03 | 0.018 | 0.27 | <0.001 | 1.06 | NS | 0.02 |
| 10 | NS | 0.23 | NS | 0.17 | 0.021 | 0.42 | 0.049 | 0.28 | <0.001 | 0.83 | <0.001 | 0.72 |
| 11 | NS | 0.06 | 0.023 | 0.29 | 0.020 | 0.41 | NS | 0.12 | 0.007 | 0.42 | NS | 0.19 |
| 12 | NS | 0.11 | 0.038 | 0.26 | 0.013 | 0.33 | NS | 0.08 | <0.001 | 0.87 | <0.001 | 0.86 |
| 13 | NS | 0.10 | 0.048 | 0.25 | NS | 0.02 | NS | 0.28 | NS | 0.25 | NS | 0.41 |
| 14 | NS | 0.30 | NS | 0.17 | NS | 0.22 | NS | 0.33 | <0.001 | 0.82 | <0.001 | 0.99 |
| 15 | NS | 0.005 | 0.001 | 0.46 | <0.001 | 0.57 | NS | 0.13 | <0.001 | 0.96 | 0.018 | 0.39 |
| 16 | 0.029 | 0.34 | 0.001 | 0.46 | NS | 0.44 | NS | 0.03 | 0.003 | 0.46 | 0.006 | 0.52 |
| 17 | NS | 0.10 | NS | 0.31 | NS | 0.04 | NS | 0.33 | <0.001 | 0.56 | 0.001 | 0.51 |
| 18 | 0.012 | 0.42 | <0.001 | 1.02 | <0.001 | 0.80 | NS | 0.22 | 0.01 | 0.45 | NS | 0.11 |
| 19 | 0.009 | 0.64 | 0.001 | 0.44 | 0.024 | 0.30 | 0.041 | 0.17 | NS | 0.09 | NS | 0.08 |
| 20 | NS | 0.16 | <0.001 | 0.63 | 0.008 | 0.60 | NS | 0.04 | NS | 0.24 | NS | 0.10 |
| 21 | 0.002 | 0.51 | <0.001 | 0.67 | 0.003 | 0.47 | 0.030 | 0.29 | <0.001 | 0.96 | NS | 0.01 |

A1: Albacete, 2^nd^ at the beginning; A2: Albacete, 2^nd^ at the end; A3: Albacete, 6^th^;

S1: Sevilla, 2nd ; S2: Sevilla, 6th

NS: no statistically significant differences
